# Extent of Inclusion of “Rural” in Comprehensive Cancer Control Plans in the United States

**DOI:** 10.5888/pcd18.210091

**Published:** 2021-09-02

**Authors:** Cathryn Murphy, Sydney Evans, Natoshia Askelson, Jan M. Eberth, Whitney E. Zahnd

**Affiliations:** 1 College of Arts and Sciences, University of South Carolina, Columbia, South Carolina; 2 Rural & Minority Health Research Center, Arnold School of Public Health, University of South Carolina, Columbia, South Carolina; 3 College of Liberal Arts & Sciences, University of Iowa, Iowa City, Iowa; 4 Department of Community and Behavioral Health, College of Public Health, University of Iowa, Iowa City, Iowa; 5 Department of Epidemiology and Biostatistics, Arnold School of Public Health, University of South Carolina, Columbia, South Carolina

## Abstract

**Introduction:**

The National Comprehensive Cancer Control Program requires states, territories, and tribal organizations to develop comprehensive cancer control plans (CCCPs). In 2019, the National Advisory Committee on Rural Health and Human Services released a series of policy recommendations, including one recommending that CCCPs address rural cancer disparities. The objective of our study was to assess the extent to which jurisdictions considered “rural” in their CCCPs.

**Methods:**

We reviewed the 66 CCCPs available on the Centers for Disease Control and Prevention’s website as of January 2020 to assess their inclusion of rural across 7 elements: 1) cancer burden data, 2) reduction of cancer disparities, 3) rural population description, 4) rural definition, 5) goals, 6) objectives, and 7) strategies. We summarized these elements by plan type (state or territory/tribal organization). For state CCCPs, we also compared the number of element types and the inclusion of rural-specific strategies by the percentage of the state’s population that was rural and the rural cancer mortality rate.

**Results:**

Of 66 plans, 45 included a mention of rural in at least 1 element, including 38 of 50 state plans and 7 of 16 territory/tribal organization plans. Reduction of cancer disparities was the most common element noted. Less than one-third of all CCCPs included a rural-specific strategy. States with a high rural cancer mortality rate tended to have at least 1 rural-specific strategy.

**Conclusion:**

Technical and financial support to improve rural data inclusion and implementation of rural-specific strategies in CCCPs may help improve the inclusion of rural data and strategy development.

**Summary**

**What is already known on this topic?**

States, territories, and tribal organizations are required to develop comprehensive cancer control plans that describe the cancer burden and disparities in their jurisdiction and provide goals, objectives, and strategies to address cancer.

**What is added by this report?**

About two-thirds of states, territories, and tribal organizations considered “rural” in their plans; only about one-third of plans included a rural-specific strategy.

**What are the implications for public health practice?**

These findings suggest that additional financial resources and technical assistance are needed to help jurisdictions address rural cancer disparities more comprehensively.

## Introduction

Rural populations, which comprise 15% to 20% of the US population, consistently have higher cancer mortality rates than urban populations (1,2). People in rural populations are often diagnosed with cancer at a more advanced stage of disease (2). They also have higher incidence rates of cancers that have mechanisms for primary and secondary prevention, such as lung, colorectal, and human papillomavirus–associated cancers (3). Factors that contribute to these disparities include high rates of poverty, lack of health insurance, and lack of access to primary and specialty care (4–8).

Recognizing the disproportionate burden of cancer in rural areas, the National Advisory Committee on Rural Health and Human Services (NACRHHS) issued a report in 2019 focused on addressing rural cancer prevention and control (9). One policy recommendation that emerged from the report was for the Centers for Disease Control and Prevention (CDC) to require US states, territories, and tribal organizations to assess and address the cancer mortality burden in their rural populations through their comprehensive cancer control plans (CCCPs) (10). States, territories, and tribal organizations are required to develop a CCCP every 5 years. CDC guidance suggests that plans present data on the jurisdiction’s cancer burden, address cancer disparities, and develop goals, objectives, and strategies to address cancer burden (11).

Despite the NACRHHS recommendation, the inclusion of rural-specific data, goals, objectives, and strategies in CCCPs has yet to be evaluated. Therefore, our objective was to analyze the extent to which the rural cancer burden is identified, contextualized, and addressed in CCCPs. Establishing this baseline is important for CDC to guide targeted recommendations for states, territories, and tribal organizations in the development of their next cancer control plans.

## Methods

We used the National Comprehensive Cancer Control Program website to access all CCCPs posted on the site as of January 2020 (10). From the website, we identified 66 CCCPs (50 states and 16 territories and jurisdictions or tribal organizations). Two reviewers (C.M. and S.E.) independently reviewed each plan for their inclusion of keywords related to rural: “rural,” “metro,” “nonmetro,” “urban,” “frontier,” “remote,” “distance,” “Appalachia,” and “Delta.” We modified this list from a previous analysis of rural consideration in cancer disparities studies that used national, population-based surveys (12). Hereinafter, we refer to the inclusion of these keywords as “rural mentions.” For each keyword appearance, we read the surrounding text to understand its context and designate whether it discussed rural communities specifically. If a keyword did not correspond to the actual mention of rural communities, we excluded it from our analysis. If 2 keywords appeared contextually in the same discussion point of rural communities, we consolidated multiple keywords into a singular rural mention.

### Coding

Once we identified rural mentions for each CCCP, we coded them into 5 categories: 1) data on cancer burden, 2) goals, 3) objectives, 4) strategies, and 5) reduction of cancer disparities. These categories are based on the Cancer Plan Index as outlined by Rochester and colleagues (13). For the purposes of this study, data on cancer burden refers to a plan’s rural-specific mention of cancer incidence, staging, and/or mortality, including a mention of a rural-specific rate or an indication of a rural–urban difference in a rate or percentage. We also coded for a description of the rural population and an indicator of how “rural” was defined in the plan. A definition of “rural” can help provide consistency in measuring rural cancer disparities, burden, and progress toward rural-specific goals, objectives, and strategies. The 2 reviewers coded each rural mention using the 7 codes and discussed data discrepancies with another member of the study team (W.Z.) to reach a consensus on the proper element for each rural mention.

The 7 codes were grouped into descriptive and action-oriented elements. The descriptive elements included discussion of the reduction of cancer disparities, data on cancer burden, description of the rural population, and an indicator of how “rural” was defined in the plan. The action-oriented elements included goals, objectives, and strategies.

### Analysis

From the data collected, we created a unified data set that summarized the rural mentions across the 7 descriptive and action-based elements overall, by state, and by territory/tribal organization. We analyzed states and territory/tribal organizations separately because states may have a more robust public health infrastructure, greater localized access to data on cancer burden, and federally derived rural definitions compared with territories and tribal organizations. For the 50 states, we performed additional comparisons: 1) a comparison of the percentage of the state population that live in rural counties (ie, nonmetropolitan) and 2) a comparison of rural age-adjusted cancer mortality rates. Nonmetropolitan counties were determined according to an Office of Management and Budget code of 5 or 6 (micropolitan or noncore) from the National Center for Health Statistics’ Urban–Rural Classification Scheme for Counties (14). Age-adjusted cancer mortality rates (2013–2017) were obtained from the National Cancer Institute, Surveillance, Epidemiology, and End Results (SEER) Program, which includes NCHS mortality data (15). We categorized the percentage of the state population living in rural counties and age-adjusted cancer mortality rates into tertiles. For populations living in rural counties, tertiles were low, 0%–12.6%; middle, 12.7%–32.3%; and high, 32.4%–69.5%. For mortality rates (per 100,000 population), tertiles were low, 131.6–158.8; middle, 158.9–179.1; and high, 179.2–211.2. Across tertiles of rural population and rural cancer burden, we compared the number of rural-specific elements and the inclusion of any rural-specific element related to reduction of cancer disparities, data on rural cancer burden, and rural-specific strategies. We also developed a series of choropleth maps using ArcGIS version 10.6 (Esri) to show the geographic distribution of rural inclusion in CCCPs overall and relative to the percentage of the state population that lived in rural counties and to the rural age-adjusted cancer mortality rates.

## Results

Forty-five of the 66 CCCPs reviewed included a rural mention in at least 1 element, including 38 of 50 states and 7 of 16 territory/tribal organization plans (Table 1). The most common type of element was related to reduction of cancer disparities, which was addressed by 33 of 66 plans overall, 29 of 50 state plans, and 4 of 16 territory/tribal organization plans. Data on cancer burden was mentioned by 18 state CCCPs. Of 66 plans overall, only 4 mentioned how rural was defined and only 11 provided a description of the rural population. Of the action-oriented elements, rural-specific strategies were the most mentioned, included in 20 plans overall, 18 state plans, and 2 territory/tribal organization plans. Only 2 states (Washington and New Mexico) included a goal focused on addressing cancer burden. No territory/tribal organization plan included a rural-specific goal, and only 4 states included a rural-specific objective.

**Table 1:** Frequency of Rural Mentions Across 7 Elements in 66 Comprehensive Cancer Control Plans, United States, January 2020

Element	Definition	Example	All Plans (N = 66)	States (n = 50)	Territories/Tribal Organizations (n = 16)
Number of plans with ≥1 element	—	—	45	38	7
Mean no. of elements (possible range, 0–7)	—	—	1.4	1.7	0.5
**Descriptive elements**					
Reduction of cancer disparities	A contributor to disparities including access to care, social determinants of health, and health care utilization	Texas: Other barriers to care arise from geographic location (particularly for those who live in border, rural, or frontier counties), immigrant status, whether one is employed in seasonal work, and one’s level of English language fluency.	33	29	4
Data on cancer burden	Statistics related to cancer incidence, staging, and/or mortality in rural areas	Florida: Deaths from cancer are lower in more populated counties such as South Florida, but higher in less populated counties in the Florida Panhandle.	18	17	1
Definition of “rural” was provided	What geographic area within the state is considered rural?	Oregon: The population of Oregon’s 36 counties is designated as frontier, rural, or urban.	4	4	0
Description of the rural population	Who is the rural population?	Alaska Native Tribal Health Consortium: Alaska Native cancer patients living in rural Alaska rely on traditional animal and plant foods gathered from the land and sea.	11	10	1
**Action-oriented elements**					
Goals	Overarching rural relevant goal	Washington: Improve access to quality, affordable, and integrated health care that incorporates routine clinical preventive services and is available in rural and urban communities alike, by effectively and strategically partnering with the health care system.	2	2	0
Objectives	Specific measurable objective proposed to achieve a specific goal	Arkansas: By 2020, increase the number of women living in rural communities who have received breast cancer screening and diagnostic services and appropriate treatment.	4	4	0
Strategies	Specific approach to meeting an objective that corresponds to a related goal	Kentucky: Increase free or low-cost transportation and housing options for persons in remote areas who must travel for treatment services.	20	18	2

State CCCPs had a similar mean number of rural-specific plan elements, regardless of the percentage of population that lived in rural counties (Table 2). The proportion of plans that described the reduction of cancer disparities and provided data on cancer burden were similar across rural population tertiles. Only 2 plans from the 17 states in the tertile with the greatest percentage of people living in rural counties provided data on rural-specific cancer burden. However, this tertile had the highest proportion of plans that included a rural-specific strategy (8 of 17).

**Table 2:** Frequency of Rural Mentions Across 4 Elements in 50 State Comprehensive Cancer Control Plans, by Percentage of Population Living in Rural Counties, United States, January 2020^a^

Element	Percentage of Population in Rural Counties		
	Low (n = 17)	Middle (n = 16)	High (n = 17)
Mean no. (SD) of rural-specific cancer plan elements (possible range, 0–7)	1.6 (1.1)	1.6 (1.4)	1.8 (1.2)
Included rural-specific cancer plan element	13	11	14
Described reduction of cancer disparities	10	8	11
Included data on cancer burden	8	7	2
Included a rural-specific strategy	4	6	8

a The 50 states were categorized into tertiles, according to percentage of population living in rural counties: low (0%–12.6%), middle (12.7%–32.3%), and high (32.3%–69.4%).

**Figure 1:**
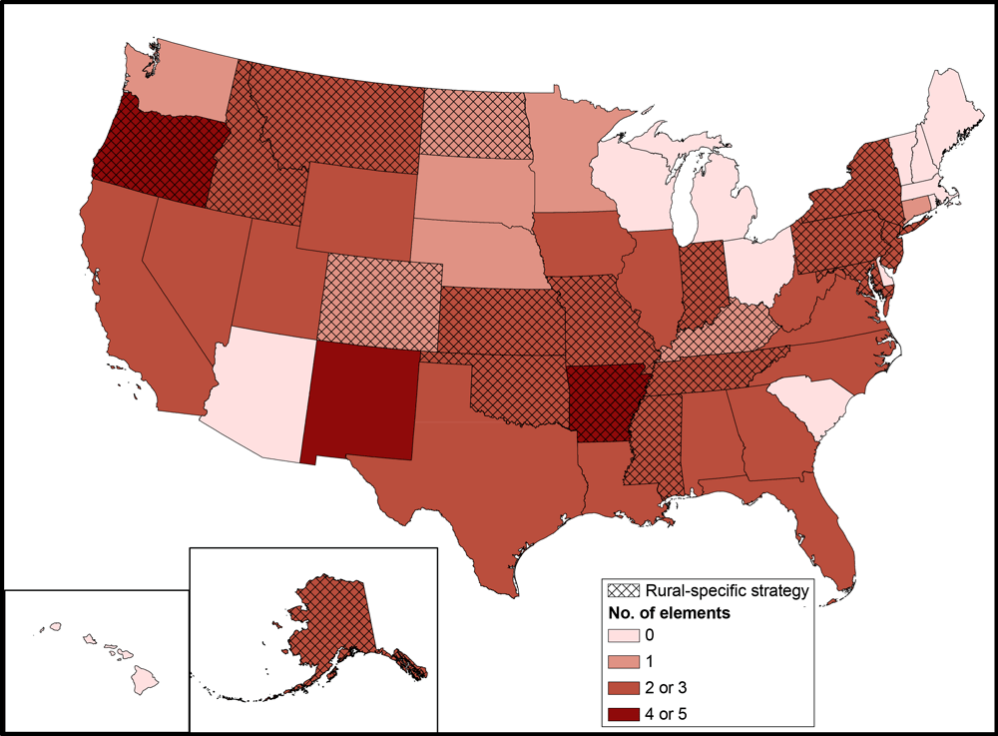
Number of elements included in cancer control plans and whether plan included a rural-specific strategy, by state. Plans were assessed as to their inclusion of “rural” across 7 elements: 1) data on cancer burden, 2) reduction of cancer disparities, 3) rural population description, 4) rural definition, 5) goals, 6) objectives, and 7) strategies. Data source: National Comprehensive Cancer Control Program, Centers for Disease Control and Prevention.

The mean number of rural-specific elements ranged from 1.5 to 2.0 across tertiles of cancer mortality rate (Table 3). Of the 47 states with rural counties, 37 had at least 1 rural-specific element across tertiles. Similar proportions of plans mentioned rural cancer disparities across tertiles. The tertile with the greatest rural cancer mortality rates had the highest proportion of including data on rural cancer burden (8 of 15) and rural-specific strategy (7 of 15). Only Arkansas (4 elements), New Mexico (5 elements), and Oregon (4 elements) had 4 or more of the 7 elements in their plans (Figure 1).

**Table 3:** Frequency of Rural Mentions Across 4 Elements in 47 State Comprehensive Cancer Control Plans, by Rural Cancer Mortality Rate, United States, January 2020^a^

Element	Rural Age-Adjusted Cancer Mortality Rate		
	Low (n = 16)	Moderate (n = 16)	High (n = 15)
Mean no. (SD) of rural-specific cancer plan elements (possible range, 0–7)	1.5 (1.1)	1.7 (1.4)	2.0 (1.1)
Included rural-specific cancer plan element	13	11	13
Described reduction of cancer disparities	9	10	10
Included data on cancer burden	2	7	8
Included a rural-specific strategy	5	5	7

a Forty-seven states were categorized into tertiles, according to rural age-adjusted cancer mortality rate per 100,000 population: low (131.6–158.8), middle (158.9–179.1), and high (179.2–211.2). Three states (New Jersey, Rhode Island, and Delaware) were not included because they had no nonmetropolitan counties.

**Table:** 

State	No. of elements	Rural-specific strategy
Alabama	2	No
Alaska	2	Yes
Arizona	0	No
Arkansas	4	Yes
California	2	No
Colorado	1	Yes
Connecticut	1	No
Delaware	0	No
Florida	2	No
Georgia	2	No
Hawaii	0	No
Idaho	3	Yes
Illinois	2	No
Indiana	2	Yes
Iowa	2	No
Kansas	3	Yes
Kentucky	1	Yes
Louisiana	3	No
Maine	0	No
Maryland	3	Yes
Massachusetts	0	No
Michigan	0	No
Minnesota	1	No
Mississippi	3	Yes
Missouri	2	Yes
Montana	2	Yes
Nebraska	1	No
Nevada	3	No
New Hampshire	0	No
New Jersey	2	Yes
New Mexico	4	No
New York	3	Yes
North Carolina	2	No
North Dakota	1	Yes
Ohio	0	No
Oklahoma	2	Yes
Oregon	5	Yes
Pennsylvania	2	Yes
Rhode Island	0	No
South Carolina	0	No
South Dakota	1	No
Tennessee	2	Yes
Texas	2	No
Utah	2	No
Vermont	0	No
Virginia	2	No
Washington	1	No
West Virginia	3	No
Wisconsin	0	No
Wyoming	3	No

**Figure 2:**
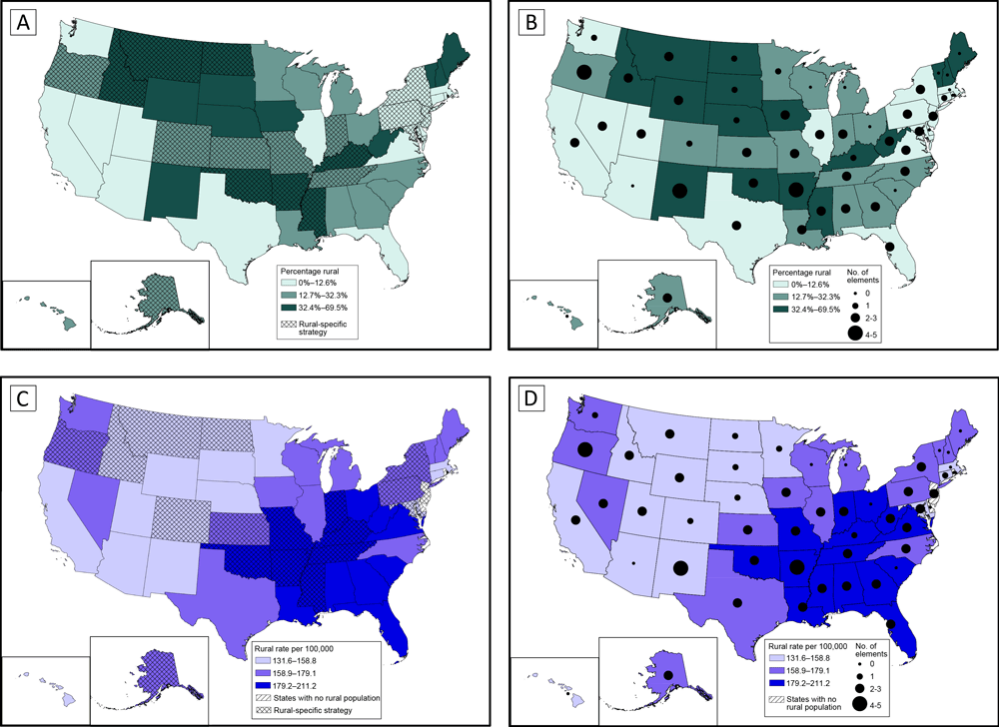
Percentage of state population residing in rural counties, rural cancer mortality rate, and inclusion of rural elements in comprehensive cancer control plan (CCCP), by state. A, Percentage of state population living in rural counties, in tertiles, and number of rural elements in state CCCP. B, Percentage of state population living in rural counties, in tertiles, and whether rural-specific strategy included in state CCCP. C, Age-adjusted cancer mortality rate per 100,000 in rural counties, in tertiles (2013–2017), and number of elements addressed in state CCCP. D, Age-adjusted cancer mortality rate per 100,000 in rural counties, in tertiles (2013–2017), and whether rural-specific strategy included in state CCCP.

States in the high tertile of rural population tended to be in northern New England, the Northern Plains, and the West, whereas states in the middle tertile of rural population were in the Midwest and South (Figure 2A and 2B). Seven of the 16 states in the high tertile of rural population had at least 1 rural-specific strategy in their CCCP, particularly states in the Northern Plains and the mid-South (Figure 2A). Many states in the middle tertile of rural population did not have a rural-specific strategy, whereas 4 states in the low tertile of rural population had rural-specific strategies. Three states in northern New England in the high tertile of rural population had no rural elements in their CCCP (Figure 2B). Of the 17 states in the high tertile of rural population, most included at least 2 rural elements.

**Table:** 

State	No. of Elements	Rural-Specific Strategy	% of Population That Lives in Rural Counties	Tertile of % Rural (Low, Middle, High)^a^	Age-Adjusted Cancer Mortality Rate per 100,000 in Rural Counties	Tertile of Cancer Mortality Rate (Low, Middle, High)^b^
Alabama	2	No	23.7	Middle	182.9	High
Alaska	2	Yes	32.4	High	165.6	Middle
Arizona	0	No	5.1	Low	134.0	Low
Arkansas	4	Yes	38.3	High	191.2	High
California	2	No	2.2	Low	157.0	Low
Colorado	1	Yes	12.7	Middle	131.6	Low
Connecticut	1	No	5.1	Low	143.3	Low
Delaware	0	No	0	Low	—	—
Florida	2	No	3.5	Low	190.3	High
Georgia	2	No	17.4	Middle	179.6	High
Hawaii	0	No	18.8	Middle	141.2	Low
Idaho	3	Yes	33.4	High	154.3	Low
Illinois	2	No	11.5	Low	179.1	Middle
Indiana	2	Yes	22.2	Middle	180.7	High
Iowa	2	No	41.0	High	165.6	Middle
Kansas	3	Yes	32.3	Middle	170.6	Middle
Kentucky	1	Yes	41.4	High	211.2	High
Louisiana	3	No	16.4	Middle	195.6	High
Maine	0	No	41.0	High	177.5	Middle
Maryland	3	Yes	2.5	Low	155.8	Low
Massachusetts	0	No	1.5	Low	153.7	Low
Michigan	0	No	18.1	Middle	172.9	Middle
Minnesota	1	No	22.5	Middle	157.7	Low
Mississippi	3	Yes	54.1	High	193.8	High
Missouri	2	Yes	25.5	Middle	185.6	High
Montana	2	Yes	64.7	High	153.1	Low
Nebraska	1	No	35.3	High	155.3	Low
Nevada	3	No	9.4	Low	163.7	Middle
New Hampshire	0	No	37.2	High	161.3	Middle
New Jersey	2	Yes	0	Low	—	—
New Mexico	4	No	33.1	High	149.1	Low
New York	3	Yes	7.1	Low	165.8	Middle
North Carolina	2	No	21.9	Middle	172.5	Middle
North Dakota	1	Yes	50.4	High	150.4	Low
Ohio	0	No	20.4	Middle	181.2	High
Oklahoma	2	Yes	34.5	High	189.3	High
Oregon	5	Yes	16.3	Middle	175.3	Middle
Pennsylvania	2	Yes	11.6	Low	171.7	Middle
Rhode Island	0	No	0.0	Low	—	—
South Carolina	0	No	15.3	Middle	184.1	High
South Dakota	1	No	52.2	High	158.8	Low
Tennessee	2	Yes	22.7	Middle	195.3	High
Texas	2	No	11.1	Low	165.9	Middle
Utah	2	No	10.6	Low	132.1	Low
Vermont	0	No	65.3	High	166.6	Middle
Virginia	2	No	12.6	Low	184.4	High
Washington	1	No	10.1	Low	161.2	Middle
West Virginia	3	No	38.4	High	189.8	High
Wisconsin	0	No	26.0	Middle	166.1	Middle
Wyoming	3	No	69.4	High	138.3	Low

^a^ Low, 0%–12.6%; middle, 12.7%–32.3%; high, 32.4%–69.5%.

^b^ Per 100,000 population: low, 131.6–158.8; middle, 158.9–179.1; high, 179.2–211.2.

States in the high tertile of rural cancer morality were largely in the South and Midwest, whereas states in the low tertile of rural cancer mortality tended to be in the West (Figure 2C and 2D). The mid-South and Deep South states of Kentucky, Tennessee, Arkansas, Oklahoma, and Mississippi had at least 1 rural-specific strategy and were in the high tertile of rural cancer mortality burden (Figure 2C). Some states in the West and Northeast in the low or middle tertile of rural cancer mortality included rural-specific strategies in their CCCPs. Northeastern states tended to include fewer rural elements and rural-specific strategies. Most states (all but South Carolina and Ohio) in the high tertile of rural cancer mortality addressed rural in at least 1 element (Figure 2D). One state (New Jersey) with no counties classified as rural had multiple cancer plan elements and rural-specific strategies.

## Discussion

NACRHHS recommends that states, territories, and tribal organizations address the rural cancer mortality burden through their mandated CCCPs (9). We examined 66 CCCPs across states, US territories, and tribal organizations to assess how rural populations were being considered and how the rural cancer burden was being assessed. We found that more than three-fourths of CCCPs among the 50 states addressed rural in some way, and just over one-third of US territories and tribal organizations explicitly discussed rural. The most common descriptive element addressed was rural cancer disparities. Rural cancer burden, rural populations, and rural definitions were less commonly noted. Less than one-third of all CCCPs had a rural-specific strategy. We found few differences in rural element inclusion across rural population and rural cancer burden tertiles. However, the tertile with the largest rural population had the lowest proportion of plans that included data on rural cancer burden, while the tertile with the highest cancer burden had the highest proportion of plans that included data on rural cancer burden.

Most states addressed at least 1 rural element, as did some territories and tribal organizations, with rural cancer disparities being the most common element. Rural cancer burden, a key focus of the NACRHHS report and a key area of cancer quality in the Cancer Plan Index, was described in CCCPs but to a lesser extent (9,13). Also, rural density, size, location, and demographics were infrequently described and defined. Descriptive cancer plan elements overlapped to some extent, in part because of a lack of rural-specific data and an appropriate characterization of rural in the CCCPs. The myriad of rural definitions may affect the inclusion of rural data or mentions in CCCPs. More than 15 federal definitions of rural exist, and some states have their own definitions of rural, creating difficulties in defining and subsequently characterizing rural (16). If rural is defined by using common federal definitions at the county or census tract level, rural-specific data can often be obtained from public sources such as CDC Wonder and SEER*Stat (cancer mortality data), central cancer registries (cancer incidence and staging data), and BRFSS (cancer screening data) (17). Although data may not be accessible externally at small geographic levels, they may be available at the county level to enable rural-specific and regional estimates of the cancer burden for use in comprehensive cancer control planning (18).

Although half of the plans described rural cancer disparities, only about one-third presented rural cancer burden data, including a few states with a high proportion of rural residents and territories and tribal organizations. Having robust data for decision making is a critical element of success in state plans. The Northwest Portland Area Indian Health Board’s plan won an award for its efforts in reducing racial/ethnic disparities (19). This organization was able to collect primary data with interagency funding from CDC to the Indian Health Service (19). Collaboration across federal agencies was a key element of other policy recommendations from the NACRHHS rural cancer report and may also be an important means of more adequately addressing rural cancer disparities in the CCCPs (9). The need for federal collaboration may be especially great for territories and tribal organizations for whom surveillance data may not be readily available. Additionally, the National Comprehensive Cancer Control Program and its partners could provide technical assistance to state, territorial, and tribal jurisdictions on how to access or collect and analyze readily available rural-specific data on the cancer burden of their populations. These data may include incidence and staging data from their respective central cancer registries and mortality data, including rural and racial/ethnic-specific data, from the National Center on Health Statistics. Such data are critical because, in addition to the elements assessed in our analysis, evaluation is a key Cancer Plan Index indicator of cancer plan quality. Inclusion of baseline rural cancer data can help guide evaluation of the success of CCCPs’ goals, objectives, and strategies.

States with a high proportion of rural populations and substantial disparities in rural cancer mortality did not frequently address rural in their CCCPs, nor did they include any rural-specific strategies to address rural cancer disparities. Some states, such as Vermont and Wyoming, which are in the high tertile of rural population and in the low tertile of cancer burden, did not explicitly include rural in their plans. One reason for this could be that, because of their large rural populations, the descriptive and action-oriented aspects of their plans may be *implicitly* rural, even if their plans did not include any of the rural-specific verbiage we searched for. Similarly, all US territories are islands, making distance a more salient barrier to cancer care, and American Indian/Alaska Native populations disproportionately live in rural areas. These unique characteristics may mean that rural concerns are implicitly rather than explicitly stated (20). However, states that have a large rural cancer burden but no rural-specific descriptions or action plans have an opportunity to improve plans to better address rural cancer disparities. CDC developed a cancer plan self-assessment tool that provides guidance in the presentation of data on cancer burden, goals, objectives, strategies, and community partner involvement, which may help plans more comprehensively describe and address rural cancer disparities (11).

Opportunities exist for additional research and policy interventions to improve the characterization and subsequent mitigation of rural cancer disparities through CCCPs. The involvement of important partners is a key component recommended by CDC’s cancer plan self-assessment tool and is an important indicator of CCCP quality (10,13). We were not able to assess the involvement of rural partners, such as rural health care providers or public health practitioners, health coalitions in rural communities, rural faith-based organizations, and rural cancer survivors, or the creation of rural-focused workgroups in CCCPs in this study, but these are vital areas for future research. The National Comprehensive Cancer Control Program has provided technical assistance and training for CCCP development in recent years (21). However, additional material support and resources (eg, funding) could be allocated to facilitate the development, implementation, and evaluation of rural-specific items in CCCPs. Moreover, jurisdictions and organizations might feel more confident to develop rural-specific strategies if more financial support for implementation were available.

Our study had several limitations. First, we evaluated plans that were available on CDC’s website during January 2020, but these plans were developed and implemented during a wide time frame. For example, if a plan covered 2015–2020, it was likely developed in previous years (eg, 2013–2014), before the National Comprehensive Cancer Control Program was modified in 2014 to provide technical support to state and other jurisdictions’ programs and training on coalition building in 2017 (21). Furthermore, some plans may have been developed before the increased rural cancer focus from federal agencies such as CDC, the Health Resources and Services Administration, and the National Cancer Institute that has occurred since 2017 (22,23). Previous studies of the evolution of CCCP development reflected changing priorities (24). The inclusion of rural in CCCPs should continue to be examined. Furthermore, in characterizing the extent of the rural population and rural cancer burden in states, we used a county-based, federal measure that may not fully capture state data on the rural population. Although we used “Delta” and “Appalachia” in identifying rural mentions that may be applicable to some states, we recognize that other words or phrases may represent largely rural or geographically isolated regions. However, we did use a dichotomization of rural that allowed for comparability across states. Another strength is that our assessment was guided by the Cancer Plan Index, a tool for assessing CCCP quality (13).

We examined the inclusion of rural-specific descriptive and action-oriented elements in published CCCPs. CCCPs across the 50 states generally addressed rural in some manner, but territorial and tribal jurisdictions did not do so as frequently. Plans described rural cancer disparities more frequently than they described the rural cancer burden or provided rural-specific strategies. Technical support to improve rural data inclusion in CCCPs and financial support for plan implementation may help improve the description of the rural cancer burden and development of rural-specific strategies. Additional research is needed to assess the role of rural partners in CCCP development and to continue to examine the inclusion of rural across plans to assess how the increasing emphasis of rural across federal agencies is reflected in CCCPs.
